# Left–Right Consistency and Quantification of Craniofacial Asymmetry by Two Fully Automatic AI-Powered Three-Dimensional Cephalometry Systems on CBCT

**DOI:** 10.3390/diagnostics16152306

**Published:** 2026-07-23

**Authors:** Zurab Khabadze, Oleg Mordanov, Ahmad Wehbe

**Affiliations:** Department of Therapeutic Dentistry, Medical Institute, RUDN University (Peoples’ Friendship University of Russia Named after Patrice Lumumba), 6 Miklukho-Maklaya Street, Moscow 117198, Russia; khabadze-zs@rudn.ru (Z.K.); wehbe-a@rudn.ru (A.W.)

**Keywords:** artificial intelligence, cone beam computed tomography, cephalometry, craniofacial asymmetry, bilateral analysis, Bland–Altman, intraclass correlation, gonial angle, Diagnocat, orthodontics

## Abstract

**Background/Objectives:** Automatic three-dimensional (3D) cephalometry on cone beam computed tomography (CBCT) is increasingly used in orthodontic and orthognathic practice, but whether fully automatic systems reproduce bilateral measurements with equal fidelity on both sides, and whether the right–left difference they report can be trusted as a clinical measure of asymmetry, has not been studied systematically. This study aimed to quantify side-specific agreement and asymmetry quantification fidelity of two commercial AI cephalometry systems against calibrated manual 3D tracing. **Methods:** Thirty-one CBCT scans were analysed by calibrated manual tracing (InVivo software), an integrated AI module (AI InVivo), and a cloud-based system (Diagnocat). Side-specific agreement was quantified using the two-way random effects intraclass correlation coefficient [ICC(2,1)], Bland–Altman analysis, and two one-sided equivalence tests (±2°/±2 mm). Asymmetry fidelity was assessed by ICC of the automatic right–left difference (Δ = R − L) against the manual Δ, Pearson correlation, and sensitivity/specificity for detecting clinically relevant asymmetry. **Results:** Per-side agreement closely mirrored overall accuracy; any systematic error was applied almost equally to both sides (bias difference ≤0.3° or ≤0.9 mm between sides). The mandibular plane angle achieved excellent ICC (0.95–0.97) and equivalence on both sides for both systems. The gonial angle was equivalent for AI InVivo (ICC 0.92–0.93) but not for Diagnocat (ICC 0.40; bias −6.95° and −6.97° on the right and left, respectively). Asymmetry agreement was generally weaker than per-side accuracy. Diagnocat showed no correlation with manual gonial asymmetry (ICC −0.12), while AI InVivo demonstrated poor agreement for body length asymmetry (ICC 0.18). Both systems under-detected clinically relevant gonial asymmetry (sensitivity 0.00–0.17), with errors predominantly in the false-negative direction. **Conclusions:** Fully automatic AI-powered 3D cephalometry applies systematic errors symmetrically to both sides, so per-side accuracy holds equally for right and left measurements. However, asymmetry fidelity is a separate, more demanding property that does not follow from absolute accuracy and remains unreliable for landmark-sensitive parameters such as the gonial angle. Clinician verification is required before automatic analysis is used for asymmetry assessment.

## 1. Introduction

Since Broadbent and Hofrath standardised the lateral cephalogram in the 1930s, cephalometric analysis has remained a routine part of orthodontic and orthognathic practice, providing a reproducible description of the skeletal and dentoalveolar relationships on which diagnosis and treatment planning are based [1,2]. The analysis rests on a small set of anatomical landmarks, and because every angle and distance is derived from where those points are placed, the reliability of the whole analysis is governed by how consistently the landmarks can be located [3,4]. Manual tracing, still treated by most authors as the reference method, is slow and operator-dependent, and intra- and inter-examiner disagreement in landmark identification is well documented [2,5,6].

Two developments have reshaped the field. The first is the shift from two-dimensional projection radiography to three-dimensional imaging with cone beam computed tomography (CBCT). A lateral cephalogram collapses a three-dimensional object onto one plane, superimposing the right and left sides so that any real side-to-side difference cannot be read directly [6,7,8]. CBCT removes this constraint and allows each side to be measured separately, which is what makes a quantitative appraisal of craniofacial asymmetry feasible in routine work [9,10]. The second development is the use of artificial intelligence (AI)—in practice, convolutional neural networks—for automatic landmark detection [2,11,12]. Automatic analysis is operator-independent and exactly repeatable for a given image, and detection times well under a minute make it attractive both for everyday diagnosis and for processing large research datasets [13,14].

The accumulated evidence is more nuanced than the early reports suggested. Across reviews of two- and three-dimensional automatic analysis, agreement with manual tracing is consistently good for dental inclinations but uneven for skeletal and soft-tissue measurements; the apical-base angles, the mandibular-plane angle and, above all, the gonial angle deviate most, often beyond the conventional two-degree or two-millimetre limits [15,16,17]. A second point runs through the recent literature and is easy to miss—reproducibility is not accuracy. Many validation studies report intraclass correlation coefficients, which describe internal consistency rather than the size of the error against the manual reference, so a system can look excellent on this index and still misplace a measurement by a clinically relevant amount [6,15]. The gonion makes the difficulty concrete. It is a constructed point, obtained by bisecting the mandibular and ramus tangents, and both manual and automatic methods place it inconsistently [15,18,19]. Where automatic landmarking has been performed directly on CBCT, the few available comparisons suggest it is, if anything, less accurate than on two-dimensional images, with the mandibular landmarks—gonion, gnathion and pogonion—showing the widest scatter [6,20,21].

What almost none of this work addresses is the bilateral behaviour of automatic analysis. With very few exceptions, validation has been carried out on a single value per patient: a unilateral measurement, or a quantity referred to an automatically defined mid-sagittal plane [6,22]. Whether a system reproduces the right and left sides with the same fidelity, and whether the right–left difference it reports can be trusted as a measure of asymmetry, has not been studied as a question in its own right. The distinction matters because asymmetry is a small quantity obtained by subtracting two larger, individually error-prone measurements. A method may agree closely with the manual reference for the conventional, averaged or single-side value and still misrepresent the difference between the sides; absolute accuracy and asymmetry fidelity need not coincide. A tool that is interchangeable with manual tracing for an angle may over- or under-state the asymmetry of that same angle, or miss the patients whose asymmetry is clinically meaningful.

Our group has been evaluating the same two commercial platforms on a common set of CBCT volumes [23,24]. An initial report compared Diagnocat with manual tracing for skeletal and dental parameters [23]; a further analysis examined dental parameters across all three methods [24]; and an agreement-and-equivalence study quantified overall accuracy for the skeletal and vertical parameters, but used only the right-side value for the bilaterally defined measurements and deferred side-specific agreement and craniofacial asymmetry to a separate analysis. The present study is that analysis. On the same volumes, and with calibrated manual three-dimensional tracing as the reference, it compares two fully automatic systems—an AI module integrated within the manual-tracing software (AI InVivo) and a separate cloud-based cross-platform system (Diagnocat)—for the bilaterally defined parameters returned by all three methods. The work is organised around two questions: whether each system agrees with the manual reference equally well on the right and the left sides, and how faithfully each system reproduces the right–left difference and the resulting classification of clinically relevant asymmetry.

## 2. Aim and Objectives

The aim of this study was to determine whether fully automatic AI-powered three-dimensional cephalometry reproduces bilateral measurements consistently and whether the asymmetry it reports can be trusted, using calibrated manual tracing on the same CBCT volumes as the reference standard. The investigation was organised around two research questions.

1. Q1—Side-specific consistency. To quantify, separately for the right and left sides, the agreement of each automatic system (AI InVivo and Diagnocat) with the manual reference for the bilaterally defined parameters, and to establish whether the magnitude and direction of any systematic error are the same on both sides.
2. Q2—Asymmetry quantification. To determine how faithfully each automatic system reproduces the right–left difference (signed asymmetry), the asymmetry index, and the binary classification of clinically relevant asymmetry derived from the manual reference.

The principal null hypotheses were that (i) side-specific agreement with manual tracing is equal between sides within each system, and (ii) the asymmetry derived from automatic analysis is equivalent to the manually derived asymmetry within the pre-specified clinical margins. We further hypothesised that, because any shared bias cancels in the right–left difference, agreement for the asymmetry would be governed chiefly by random error and would therefore be weaker than the corresponding side-specific agreement, and that asymmetry fidelity would be parameter- and system-specific rather than a simple corollary of absolute accuracy.

## 3. Materials and Methods

Sample, imaging, and cephalometric methods. The study sample, the three cephalometric methods (calibrated manual tracing in InVivo as the reference standard; the integrated AI InVivo module; and the cloud-based Diagnocat system), and the operator-calibration procedure followed the companion agreement-and-equivalence study on the same volumes. In brief, CBCT scans of 31 consecutive patients (25 female, 6 male; mean age 29.2 ± 6.7 years, range 18–49) retained after calibration were analysed; for three patients the automated Diagnocat report could not be generated, so comparisons involving Diagnocat were based on complete cases (28 patients for angular and 27 for linear parameters). For both automated methods the operators were blinded to the manual results, and no manual adjustment of automatically detected landmarks was performed. CBCT volumes were acquired on a GALILEOS Comfort unit (Sirona Dental Systems GmbH, Bensheim, Germany) with the following settings: tube voltage 98 kV, tube current 5 mAs, scan time 14 s, field of view 15 × 15 cm, and isotropic voxel size 0.25 mm. All scans were obtained under identical acquisition settings; patients were positioned in habitual occlusion with the lips and tongue at rest, and the head was stabilised with head and chin supports. Examiner calibration followed the protocol of the companion study on these volumes [23]: a maxillofacial radiology specialist and an orthodontist each traced every volume twice, with a seven-day interval between tracings. A volume was excluded if the inter-examiner difference, or the intra-examiner difference for either specialist, exceeded half of one standard deviation of the parameter; after calibration no volume required exclusion on this criterion, indicating close inter- and intra-operator agreement for the parameters analysed here, and the mean of the calibrated tracings was used as the manual reference.

Bilateral parameters. The present analysis was restricted to parameters that are defined bilaterally and were returned for both sides by all three methods: the mandibular plane angle to SN, the gonial angle (both in degrees), and ramus length and mandibular body length (both in millimetres). The right and left occlusal plane angle and the right and left incisor inclinations were not available bilaterally from all methods (see Section 4, data completeness) and were therefore excluded from the primary across-method analysis; bilateral incisor measurements available from manual tracing and Diagnocat are reported separately as a secondary analysis.

Side-specific agreement (Q1). For each parameter and each side, agreement of each automatic system with the manual reference was quantified with the intraclass correlation coefficient based on a two-way random effects, single-measurement, absolute-agreement model [ICC(2,1)], with 95% confidence intervals computed by the method of McGraw and Wong and interpreted as poor (<0.50), moderate (0.50–0.75), good (0.75–0.90), or excellent (>0.90); with Bland–Altman analysis of bias and 95% limits of agreement; and with two one-sided tests of equivalence against a priori margins of ±2° (angular) and ±2 mm (linear). Side-dependence of bias was assessed by comparing the right- and left-side bias within each system.

Asymmetry quantification (Q2). For every patient and method the signed asymmetry was defined as the right-minus-left difference (Δ = R − L); its magnitude |Δ| and, for the linear parameters, the asymmetry index AI% = |R − L| ÷ [(R + L)/2] × 100 were also computed. The fidelity of automatic asymmetry was evaluated by the agreement of the automatic Δ with the manual Δ [ICC(2,1), Pearson correlation, Bland–Altman bias and limits of agreement]. Patients were further classified as showing clinically relevant asymmetry using illustrative thresholds of |Δ| > 4° for the angular parameters and AI% > 3% for the linear parameters; agreement of this binary classification between each system and the manual reference was summarised by sensitivity, specificity, Cohen’s κ, and the mean absolute error of the asymmetry metric. As a technical validity check, automatic outputs returning identical right and left values for a bilateral structure were recorded as failures of side differentiation. Analyses were performed in Python version 3.11 with SciPy version 1.14 (Python Software Foundation); α was set at 0.05. No prospective power calculation was performed; the sample size was fixed by the number of consecutive patients meeting the inclusion criteria. Because the sample was defined by consecutive availability rather than by an a priori calculation, inferential uncertainty is expressed throughout as the width of the 95% confidence intervals of the agreement and equivalence estimates rather than as a power figure; for the more variable parameters these intervals are correspondingly wide, and the equivalence conclusions for those parameters should therefore be read as imprecise rather than definitive.

## 4. Results

Sample and data completeness. Thirty-one patients contributed manual and AI InVivo measurements; Diagnocat contributed 28 (angular) or 27 (linear) complete cases. Two structural limitations of the automated outputs constrained the bilateral analysis and are themselves findings. First, the AI InVivo module did not return left-side values for the incisor inclinations or for the occlusal plane, and the left occlusal plane was likewise absent from the manual output; bilateral analysis of the occlusal plane across methods was therefore not possible, and bilateral incisor analysis was confined to manual tracing versus Diagnocat. Second, Diagnocat returned identical right and left values—indicating a failure of side differentiation—in four instances (one gonial angle, one ramus, one occlusal plane, and one lower-incisor case), spuriously reporting zero asymmetry for those structures; no such failure occurred with manual tracing or AI InVivo. The four parameters analysable bilaterally across all three methods were the mandibular plane angle, the gonial angle, ramus length, and body length.

### 4.1. Q1—Side-Specific Consistency

Side-specific descriptive values. Right- and left-side means were closely matched within each method (Table 1). The most conspicuous between-method discrepancy reproduced the pattern of the companion study and was the same on both sides: Diagnocat returned systematically higher gonial angles than manual tracing or AI InVivo on the right (127.7° vs. 120.8° and 119.7°) and on the left (128.0° vs. 121.3° and 120.3°).

**Figure 1:**
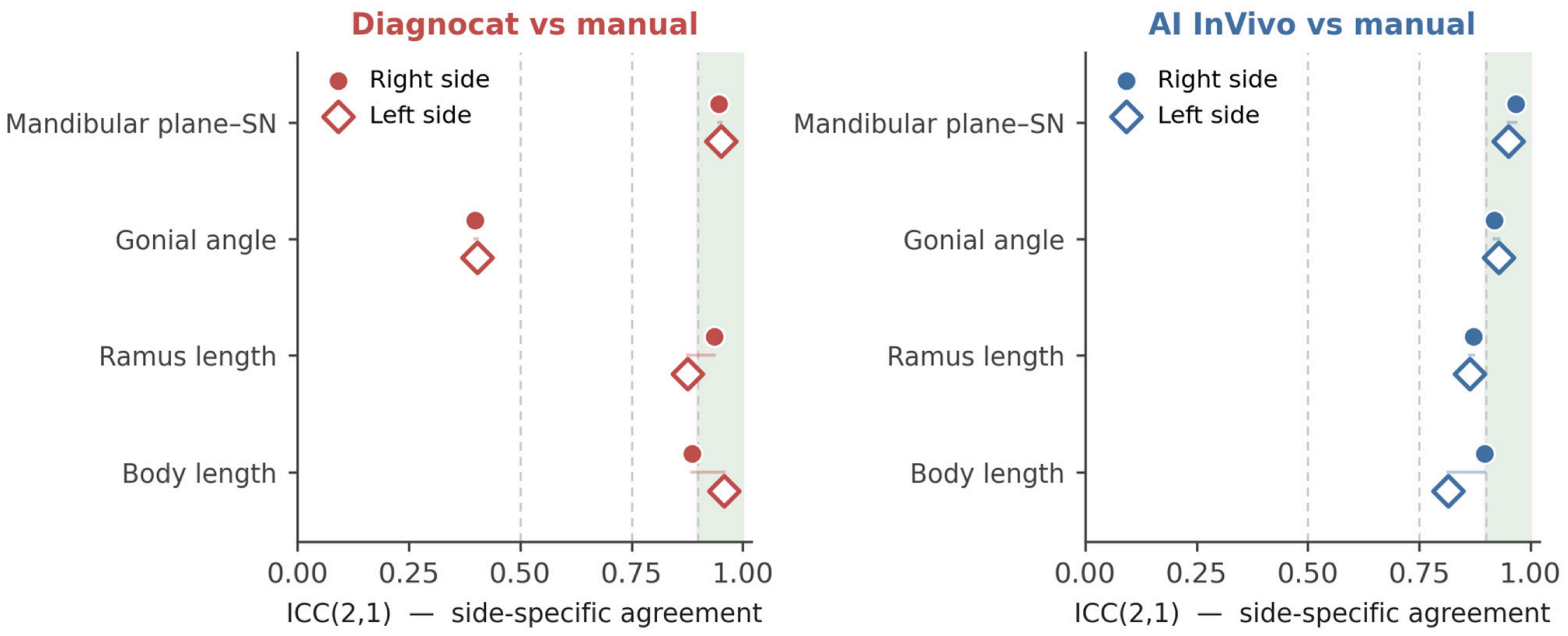
Agreement with Manual tracing is symmetric between sides (Q1). Side-specific agreement [ICC(2,1)] of each automatic system with manual tracing, shown separately for the right (circles) and left (open diamonds) sides. The shaded band marks the excellent-agreement region (ICC ≥ 0.90). Right- and left-side estimates coincide closely for every parameter, indicating that systematic error is applied symmetrically to both sides.

Side-specific agreement and side-symmetry of bias. For every parameter and system the right-side and left-side agreement with manual tracing were closely comparable (Table 2, Figure 1). Critically, the systematic errors identified in the companion study were reproduced almost identically on the two sides rather than being confined to one: Diagnocat overestimated the gonial angle by 6.95° on the right and 6.97° on the left (ICC 0.40 on both sides; equivalence not met on either), and AI InVivo underestimated ramus length by 2.07 mm on the right and 2.22 mm on the left (ICC 0.87 and 0.86; equivalence not met on either). Across the four parameters the right- and left-side bias estimates within a system differed by no more than 0.3° for the angular parameters and 0.9 mm for the linear parameters. The mandibular plane angle (ICC 0.95–0.97 for both systems and sides) and the body length (ICC 0.81–0.96) were equivalent to manual tracing on both sides for both systems; AI InVivo was equivalent for the gonial angle on both sides (ICC 0.92 and 0.93), whereas Diagnocat was not. No parameter showed a side-dependent direction of bias for either system.

### 4.2. Q2—Asymmetry Quantification

**Figure 2:**
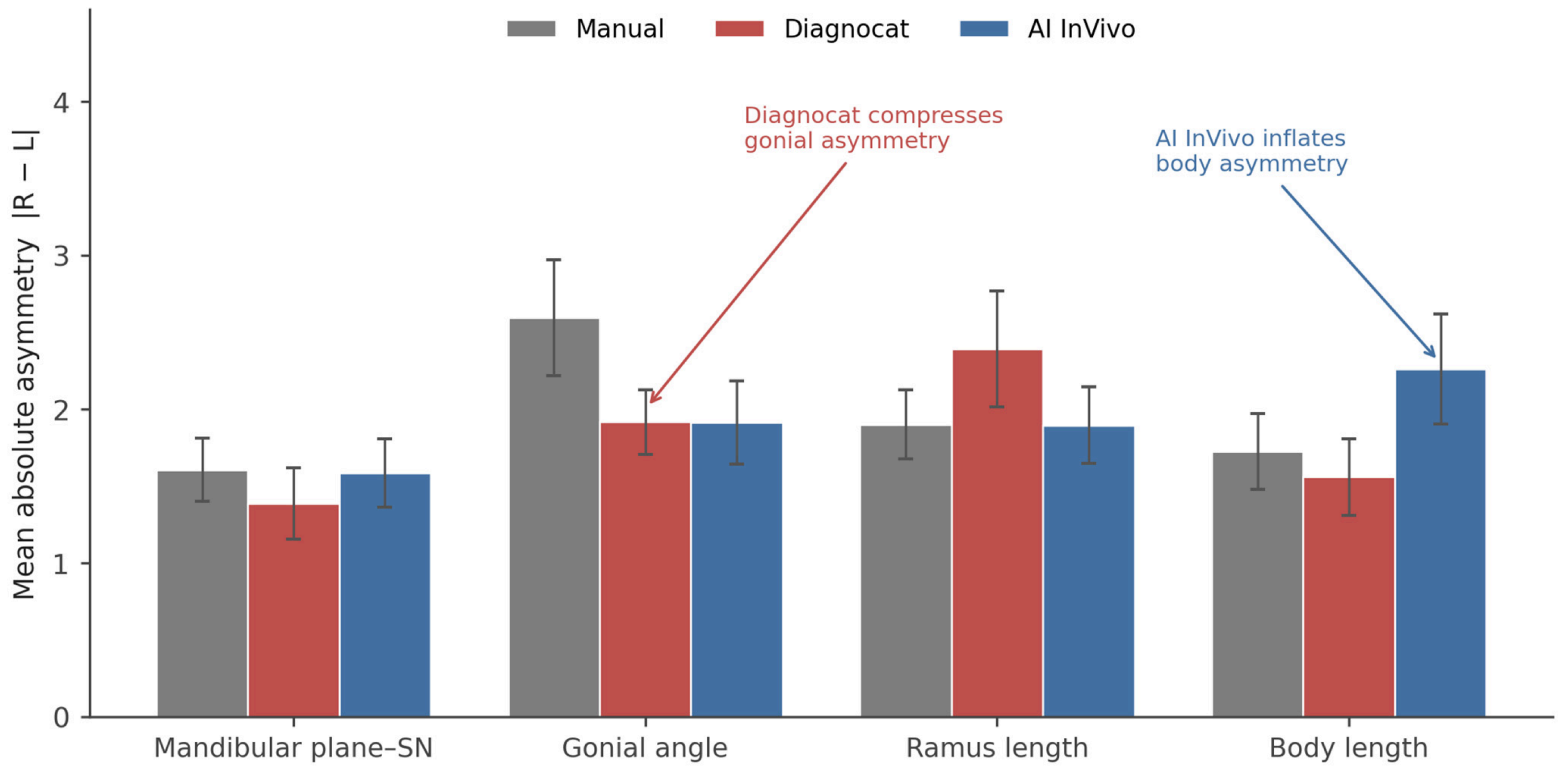
Magnitude of detected asymmetry by method (angular in °, linear in mm). Mean absolute asymmetry |R − L| by method for each parameter (error bars = SEM; angular parameters in degrees, linear in millimetres). Diagnocat compresses gonial-angle asymmetry; AI InVivo inflates body-length asymmetry.

Magnitude of detected asymmetry. Mean absolute asymmetry was small for all parameters and methods, but the two systems distorted it in opposite directions for different parameters (Table 3, Figure 2). For the gonial angle, manual tracing recorded a mean |Δ| of 2.60° with a maximum of 8.42°, whereas Diagnocat compressed the asymmetry to a mean of 1.92° and a maximum of only 3.80°, and AI InVivo to 1.91° (maximum 4.49°); both systems thus narrowed the gonial asymmetry, Diagnocat most severely, effectively truncating its range. Conversely, for body length AI InVivo inflated the asymmetry (mean |Δ| 2.26 mm, maximum 7.13 mm) relative to manual tracing (1.72 mm, maximum 5.02 mm). For the mandibular plane angle and ramus length the mean asymmetry magnitudes were similar across methods.

**Figure 3:**
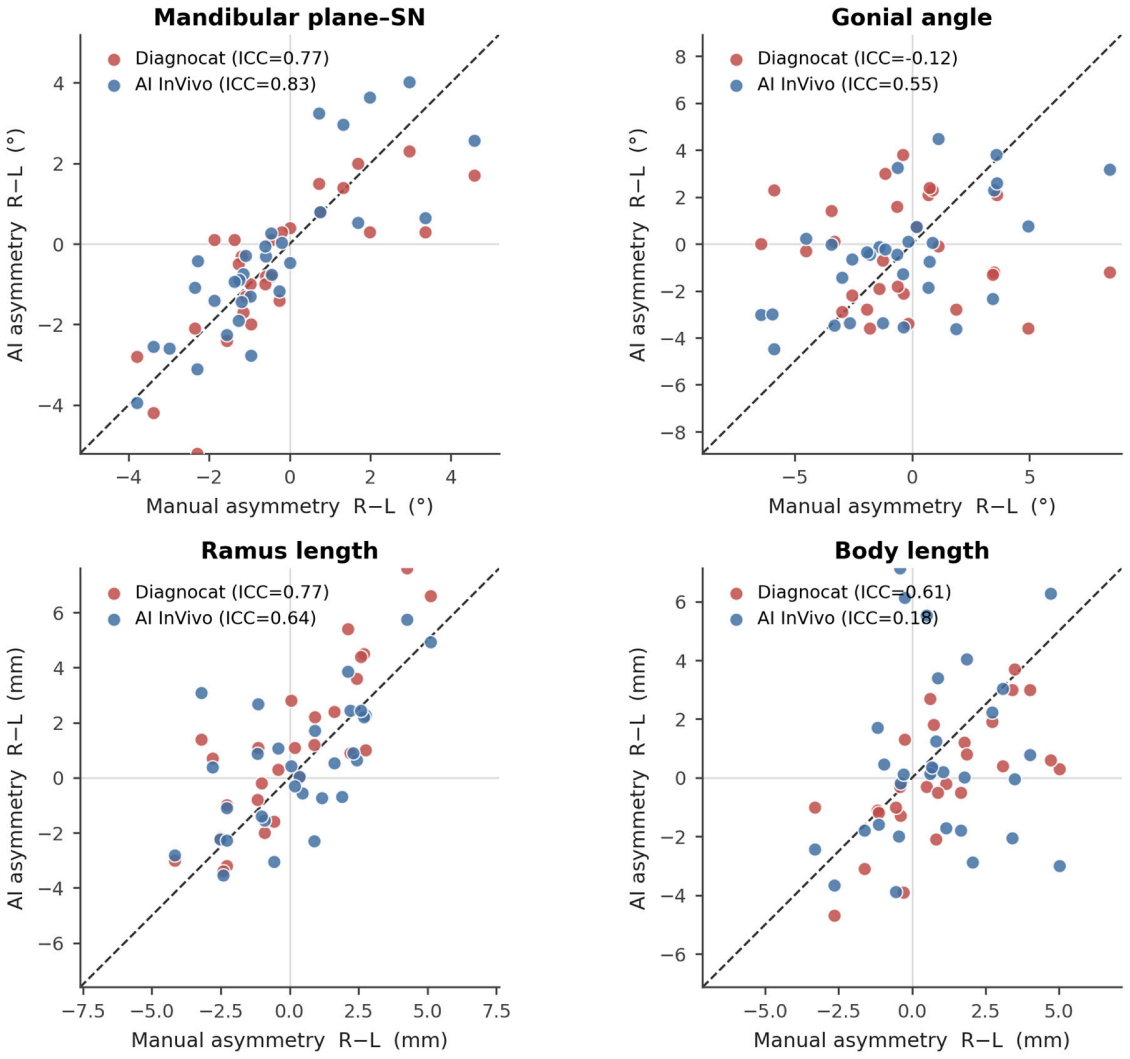
Agreement of AI- vs. manual-derived asymmetry (Δ = R − L) by parameter (Q2). Automatic versus manual asymmetry (Δ = R − L) for each parameter, with the line of identity. Points on the diagonal indicate faithful reproduction of asymmetry. The gonial angle panel shows the Diagnocat points (red) forming a near-horizontal cloud—no relationship with the manual asymmetry—whereas AI InVivo (blue) follows the diagonal more closely; for body length the dispersion of the AI InVivo points reflects its low concordance.

**Fidelity of automatic asymmetry.** Agreement between the automatic and manual asymmetry (Δ = R − L) was, as predicted, generally weaker than the corresponding side-specific agreement and varied markedly by parameter and system (Table 3, Figure 3). It was good for the mandibular plane angle (ICC 0.77 for Diagnocat and 0.83 for AI InVivo). For the gonial angle the two systems diverged sharply: AI InVivo achieved only moderate agreement (ICC 0.55), while Diagnocat showed no correlation with the manual asymmetry at all (ICC −0.12, r −0.12), so that its right–left difference carried essentially no information about the true gonial asymmetry—a result unchanged after exclusion of the single side-differentiation failure. For ramus length agreement was good for Diagnocat (ICC 0.77) and moderate for AI InVivo (ICC 0.64), whereas for body length the ordering reversed, agreement being moderate for Diagnocat (ICC 0.61) but poor for AI InVivo (ICC 0.18). No single system reproduced asymmetry reliably across all four parameters, and the system that was more accurate for the absolute gonial angle (AI InVivo) was the less reliable of the two for body length asymmetry, confirming that asymmetry fidelity does not follow from absolute accuracy.

**Classification of clinically relevant asymmetry.** The prevalence of asymmetry exceeding the illustrative thresholds, and the pattern of its detection, depended strongly on the parameter (Table 4). These classification statistics are reported as exploratory and descriptive only; the low prevalence of clinically relevant asymmetry for several parameters precludes stable estimation of Cohen’s κ, sensitivity, or specificity, and the mandibular-plane parameter (a single positive case, 1/31) has been omitted from Table 4 for this reason. For the gonial angle the manual reference flagged six patients, of whom Diagnocat identified none and AI InVivo one, the dominant error in both cases being false-negative—that is, classifying a truly asymmetric patient as symmetric. Detection was best for ramus length, where asymmetry beyond 3% was common (manual prevalence 55%) and AI InVivo showed the closest correspondence with the manual reference, Diagnocat was somewhat lower. For body length the correspondence was weaker for both systems. Within this exploratory analysis the systems erred towards under-detection of asymmetry rather than over-detection.

Secondary analysis—bilateral incisor inclination (manual versus Diagnocat). Because AI InVivo did not output left-side incisor values, bilateral incisor asymmetry could be compared only between manual tracing and Diagnocat (Table 5). Agreement of the asymmetry was excellent for the lower-incisor inclination to the mandibular plane (ICC 0.91), good for the interincisal angle (ICC 0.78), and moderate for the upper-incisor inclination to SN (ICC 0.63), with mean absolute asymmetries of comparable magnitude between the two methods.

**Overall pattern.** Both automatic systems reproduced bilateral measurements with side-symmetric accuracy; per-side agreement mirrored the overall agreement established in the companion study, and every systematic error was applied almost equally to the right and left sides, so that no side was privileged and the companion study’s right-side-only analysis did not conceal a side-dependent bias. Fidelity to the asymmetry itself was a separate and more demanding property: it was generally weaker than per-side accuracy, was parameter- and system-specific, did not follow from absolute accuracy, and—for the clinically important detection of gonial asymmetry—was unreliable for both systems, with errors falling predominantly in the false-negative direction.

## 5. Discussion

This study set out to determine whether two fully automatic systems reproduce bilateral cephalometric measurements consistently, and whether the asymmetry they report can be trusted, using calibrated manual three-dimensional tracing on the same CBCT volumes as the reference. Two results stand out. Per-side accuracy was symmetric; for every parameter and system, the agreement with manual tracing on the right side closely matched that on the left, and any systematic error was applied almost equally to both sides. Fidelity to the asymmetry itself was a different matter—generally weaker than per-side accuracy, specific to the parameter and the system, and not predictable from absolute accuracy. For the gonial angle, the measurement most often singled out as problematic in the literature, neither system reproduced the asymmetry reliably, and when the systems erred they erred towards calling an asymmetric patient symmetric.

The side-symmetry of bias is the more reassuring of the two findings and is informative about the nature of the errors. The cross-platform system overestimated the gonial angle by about seven degrees, and it did so to within a fraction of a degree on both sides; the within-software module underestimated ramus length by roughly two millimetres, again almost equally on the left and the right. Errors of this kind—uniform in direction and nearly identical between sides—are the signature of a definition-related offset rather than random imprecision, and they are consistent with the long-recognised sensitivity of the gonion to how the mandibular and ramus tangents are constructed [15,18,19]. The practical consequence is that the right-side-only approach used in our agreement-and-equivalence study did not conceal a side-dependent problem; what holds for one side holds, to a close approximation, for the other. For per-side measurement, each system behaves on the left as the companion study showed it behaves on the right.

The weaker agreement for asymmetry follows from the same arithmetic that makes per-side accuracy reassuring. When a bias is shared by both sides it cancels in the right–left difference, leaving the random component, and random errors on the two sides add rather than cancel. Asymmetry is therefore the quantity most exposed to imprecision, and it is exactly here that reproducibility and accuracy come apart; a system can return a believable gonial angle on each side yet produce a right–left difference that carries little information about the true asymmetry. The cross-platform system illustrates this most clearly. Its gonial asymmetry showed no correlation with the manual asymmetry, and its range was compressed—the largest manual side difference of more than eight degrees was flattened to under four—so the overestimation of the absolute angle was accompanied by a near-total loss of the asymmetry signal. The within-software module behaved differently again, tracking gonial asymmetry moderately well but inflating and scattering body length asymmetry. No single system was best across all four parameters, which is the practical face of the decoupling between absolute accuracy and asymmetry fidelity. The point also extends a caveat already made for landmark errors in general, namely that small deviations propagate into the derived measurements [15], to the specific case of a difference between two measurements.

Translating these differences into a binary judgement of clinically relevant asymmetry sharpened the clinical message. For the angular parameters, asymmetry beyond the chosen threshold was uncommon in the sample, and both systems tended to miss the few patients who had it, so agreement on classification was poor and the errors were predominantly false-negative. Detection was best for ramus length, where asymmetry was both common and larger, and where the within-software module reached moderate agreement with the manual reference. Under-detection is the less desirable direction of error for a screening task; a system that quietly reports symmetry where asymmetry exists is more likely to mislead than one that over-calls and prompts a second look. On the present evidence, automatic analysis can support the measurement of each side, but it should not be relied on to exclude asymmetry, least of all for the gonial angle.

The clinical weight of these errors depends on the decision they feed. The gonial angle findings are the most consequential; a systematic overestimation of roughly seven degrees, combined with a right–left difference that carries almost no information about the true asymmetry, could lead a clinician to misjudge the vertical growth pattern and, in a patient screened for mandibular asymmetry, to overlook a genuine side-to-side difference—the error direction least acceptable when the analysis is used to decide whether asymmetry warrants further imaging, orthodontic compensation, or referral for orthognathic assessment. For the linear mandibular dimensions, an inflated body length asymmetry could conversely prompt unnecessary concern. Because these are the parameters that inform decisions about asymmetric extraction patterns, asymmetric mechanics, and surgical mandibular repositioning, the practical message is that automatic asymmetry output should be verified against the images before it is allowed to influence such plans, whereas the well-agreeing parameters such as the mandibular plane angle can reasonably serve as a first pass.

Two features of the automatic outputs are findings in their own right. The within-software module returned no left-side values for the incisor inclinations or the occlusal plane, so bilateral analysis of those parameters could not be carried out across all three methods, and the cross-platform system occasionally returned identical right and left values for a bilateral structure, reporting an asymmetry of exactly zero where the manual reference and the other system found a real difference. Neither behaviour is apparent to the user from the report alone, and both reflect the proprietary, closed nature of commercial systems and the continuing absence of a validated, standardised set of three-dimensional bilateral landmarks [5,15,25]. They are a reminder that an automatic cephalometric report is not self-validating; a plausible number on each side does not guarantee that the two sides were located independently.

These two behaviours warrant closer scrutiny. The four cases in which Diagnocat returned identical right and left values all occurred in patients whose manual right–left difference for the affected parameter was among the smallest in the sample, while visual inspection of those volumes revealed no artefacts, motion, or morphological anomaly; this is consistent with the hypothesis that the system applies a symmetry-forcing behaviour when the predicted bilateral discrepancy falls below an internal threshold, rounding a small real difference to zero, rather than responding to image quality; one possible explanation is an internal rounding or tie-breaking rule, but, the pipeline being closed, this remains an inference from the observed pattern rather than a confirmed algorithmic mechanism. The absence of left-side incisor and occlusal-plane values from AI InVivo, by contrast, is a reporting limitation of the module’s exported cephalometric report in the version used rather than a failure of landmark detection within the volume. Neither behaviour is apparent to the user from the report alone, which underlines that a plausible number on each side does not certify that the two sides were located, or even reported, independently.

These results sit within the broader literature, which has repeatedly found automatic cephalometry accurate enough for screening some parameters but not others, and which recommends treating its outputs as a clinician-supervised first pass rather than an unsupervised replacement for expert tracing [5,15,26,27]. They also reinforce the distinction, increasingly emphasised in recent reviews, between statistical agreement and clinical accuracy; an analysis judged interchangeable on an intraclass correlation coefficient or a mean difference may still fail for a quantity, such as asymmetry, that those summary statistics do not directly address [6,15]. Agreement is more fully characterised when the intraclass correlation coefficient is read alongside the magnitude and direction of systematic and random error [28,29,30,31]. The cross-platform system retains the practical advantages reported previously—shorter analysis time, lower cost and the elimination of operator-dependent variability [24,32]—but the present data attach a parameter-specific caution to its use for asymmetry, and a comparable caution to the within-software module for the linear mandibular dimensions. Facial asymmetry is common in the general population and becomes clinically relevant as skeletal disproportion increases, so its objective quantification is an established goal of craniofacial diagnosis [33,34]. Two-dimensional assessment of mandibular asymmetry from panoramic radiographs is limited by projective distortion and vertical magnification, which reduce the reliability of side-to-side comparison [35,36]; three-dimensional imaging removes these constraints and has led to morphometric methods for defining the mid-sagittal plane and quantifying craniofacial asymmetry [37]. Evaluations of web-based, fully automated cephalometric platforms have reached the same practical conclusion, reporting that automatically generated measurements should be verified before clinical use [38].

Recent studies reinforce this reading. An independent validation of commercial AI cephalometric analysis on CBCT concluded that differences in the core sagittal angles were large enough to alter diagnosis and treatment planning and that AI output should be reviewed and, where necessary, corrected before clinical use [39]. A deep learning 3D-landmarking study likewise recommended manual verification of uncertain landmarks, the gonion among them, before cephalometric analysis [40], and a multicentre validation of automatic 3D landmark detection reported mean radial errors reaching several millimetres for mandibular points [41]. These converge on the present finding that per-landmark and per-parameter behaviour, and the gonion in particular, must be examined directly rather than inferred from overall accuracy.

Several limitations qualify these conclusions. The most important is the small sample size and the resulting imprecision of the estimates. The sample of 31 patients—reduced to 27–28 for the Diagnocat linear parameters owing to report generation failures—was not sized by a prospective power calculation, and the confidence intervals for the agreement and equivalence estimates are correspondingly wide, particularly for the asymmetry analyses and for the classification statistics; these results should be read as preliminary estimates requiring confirmation in adequately powered samples. Generalizability is a second constraint; all volumes were acquired on a single CBCT unit (GALILEOS Comfort) with one acquisition protocol (15 × 15 cm field of view, 0.25 mm voxel), in one centre, from a predominantly female series. The predominantly female composition reflects the sex distribution of the consecutive patient series rather than a deliberate selection criterion, and it limits the representativeness of the asymmetry prevalence estimates. Landmark visibility, and therefore automatic landmarking behaviour, depends on voxel size, field of view, dose, and reconstruction, so the error magnitudes reported here—in particular the gonial angle offset and the side differentiation failures—may differ on other devices, protocols, or populations, and the findings should be regarded as device- and sample-specific until replicated more widely. Third, calibrated manual tracing is a reference standard, not an absolute ground truth; manual landmark identification is itself subject to inter- and intra-operator variability, greatest for constructed points such as the gonion, so part of the disagreement attributed to the automatic methods reflects imprecision in the reference itself, and the agreement indices should be read as agreement between two imperfect methods [6]. The thresholds used to classify clinically relevant asymmetry are illustrative rather than established, and because asymmetry was uncommon for several parameters the corresponding classification statistics rest on few positive cases and are exploratory. Because AI InVivo did not output left-side values for the incisor inclinations or the occlusal plane, a three-way comparison across both systems and the manual reference was not possible for these parameters, which narrows the comparative scope of the study and prevents any conclusion about the bilateral fidelity of AI InVivo for dental axis measurements. Finally, the proprietary nature of both systems means that the mechanisms proposed here—a definition-related gonial offset, a failure of side differentiation—are inferred from the pattern of the data rather than confirmed against a disclosed algorithm. Larger, multi-centre and demographically balanced studies, using agreed three-dimensional landmark definitions and asymmetry thresholds and a wider range of devices, are needed to confirm and generalise these findings, and direct scrutiny of how each system constructs the gonion would help to explain the parameter that proved least trustworthy [42].

Finally, these systems are not static. Commercial AI cephalometry is updated continuously, and the specific behaviours documented here—the gonial angle offset, the compression of gonial asymmetry, and the occasional side differentiation failure—may be reduced, or altered, by future algorithm versions. Two implications follow: results such as ours are tied to a stated software version and acquisition date and should be re-validated periodically, and greater version transparency from vendors would materially aid reproducibility. Until such transparency and independent re-validation become routine, clinician verification of automatic asymmetry output remains the prudent default.

## 6. Conclusions

On a common set of CBCT volumes, both fully automatic systems reproduced bilateral measurements with side-symmetric accuracy. Per-side agreement with manual tracing mirrored the overall agreement established for the same parameters, and every systematic error was applied almost equally to the right and left sides, so no side was privileged. This supports the right-side-only approach taken in the companion agreement-and-equivalence study and indicates that, for per-side measurement, each system performs on the left as it does on the right.

Fidelity to the asymmetry itself was a separate and more demanding property. It was generally weaker than per-side accuracy, varied with the parameter and the system, did not follow from absolute accuracy, and—for the gonial angle—was unreliable for both systems, with errors falling mainly in the false-negative direction. One system lost the gonial asymmetry signal almost entirely while overestimating the angle; the other reproduced gonial asymmetry moderately but inflated the asymmetry of the mandibular body.

Fully automatic three-dimensional cephalometry can therefore support the measurement of each side, but the asymmetry it reports requires clinician verification, particularly for landmark-sensitive angles such as the gonial angle, where a system may understate or miss a real side-to-side difference. Larger multi-centre studies with agreed landmark definitions and asymmetry thresholds are needed before automatic analysis can be relied upon for the assessment of craniofacial asymmetry.

## Figures and Tables

**Table 1:** Side -specific descriptive statistics (mean ± SD) by method and side.

Parameter	Side	Manual InVivo	Diagnocat	AI InVivo
Mandibular plane–SN (°)	R	34.15 ± 6.11	32.68 ± 5.72	34.60 ± 5.64
	L	34.63 ± 5.57	33.26 ± 4.99	34.98 ± 5.01
Gonial angle (°)	R	120.82 ± 5.31	**127.66 ± 6.51**	119.72 ± 5.37
	L	121.29 ± 6.09	**128.03 ± 6.15**	120.25 ± 5.19
Ramus length (mm)	R	57.54 ± 4.99	56.34 ± 5.28	55.46 ± 5.16
	L	57.25 ± 4.82	55.23 ± 5.22	55.02 ± 5.02
Body length (mm)	R	83.98 ± 3.78	82.85 ± 4.43	83.99 ± 3.52
	L	83.11 ± 3.42	82.86 ± 3.82	83.47 ± 4.12

Manual InVivo *n* = 31; Diagnocat *n* = 27–28; AI InVivo *n* = 31.

**Table 2:** Side-specific agreement with manual tracing: ICC(2,1) [95% CI], Bland–Altman bias and 95% limits of agreement, and equivalence (±2°/±2 mm).

Parameter	System	Side	ICC [95% CI]	Bias	95% LoA	Eq.
Mandibular plane–SN	Diagnocat	R	0.95 [0.66, 0.98]	+1.38	−1.44, +4.20	Yes
	Diagnocat	L	0.95 [0.72, 0.98]	+1.11	−1.28, +3.49	Yes
	AI InVivo	R	0.97 [0.93, 0.98]	−0.46	−3.34, +2.43	Yes
	AI InVivo	L	0.95 [0.90, 0.98]	−0.35	−3.64, +2.94	Yes
Gonial angle	Diagnocat	R	0.40 [−0.11, 0.74]	−6.95	−16.18, +2.28	No
	Diagnocat	L	0.40 [−0.11, 0.74]	−6.97	−16.31, +2.38	No
	AI InVivo	R	0.92 [0.79, 0.96]	+1.09	−2.68, +4.87	Yes
	AI InVivo	L	0.93 [0.82, 0.97]	+1.04	−2.76, +4.84	Yes
Ramus length	Diagnocat	R	0.94 [0.73, 0.98]	+1.21	−1.78, +4.19	Yes
	Diagnocat	L	0.88 [0.10, 0.97]	+2.19	−0.90, +5.29	No
	AI InVivo	R	0.87 [0.20, 0.96]	+2.07	−1.28, +5.43	No
	AI InVivo	L	0.86 [0.06, 0.96]	+2.22	−0.81, +5.26	No
Body length	Diagnocat	R	0.89 [0.57, 0.96]	+1.29	−1.90, +4.48	Yes
	Diagnocat	L	0.96 [0.91, 0.98]	+0.34	−1.72, +2.40	Yes
	AI InVivo	R	0.90 [0.80, 0.95]	−0.01	−3.31, +3.28	Yes
	AI InVivo	L	0.81 [0.65, 0.91]	−0.36	−4.90, +4.17	Yes

Bias = manual − automatic; a negative bias indicates the automatic method returned the higher value. Diagnocat *n* = 27–28; AI InVivo *n* = 31. Eq. = equivalent within ±2°/±2 mm by two one-sided tests.

**Table 3:** Asymmetry magnitude by method and agreement of automatic asymmetry with manual tracing.

Parameter	Method	Signed Δ (Mean ± SD)	|Δ| Mean (Max)	Δ-Agreement vs. Manual	Pearson r	Δ Bias [95% LoA]
Mandibular plane–SN (°)	Manual	−0.48 ± 1.94	1.61 (4.59)	—	—	—
	Diagnocat	−0.58 ± 1.77	1.39 (5.20)	0.77 [0.57, 0.89]	0.78	+0.28 [−2.16, +2.71]
	AI InVivo	−0.38 ± 1.99	1.59 (4.02)	0.83 [0.68, 0.92]	0.83	−0.10 [−2.36, +2.15]
Gonial angle (°)	Manual	−0.47 ± 3.34	**2.60 (8.42)**	—	—	—
	Diagnocat	−0.36 ± 2.22	1.92 (3.80)	−0.12 [−0.49, 0.27]	−0.12	+0.02 [−8.10, +8.13]
	AI InVivo	−0.53 ± 2.40	1.91 (4.49)	0.55 [0.24, 0.76]	0.57	+0.06 [−5.40, +5.51]
Ramus length (mm)	Manual	+0.29 ± 2.28	1.90 (5.12)	—	—	—
	Diagnocat	+1.10 ± 2.91	2.39 (7.60)	0.77 [0.46, 0.90]	0.83	−0.99 [−4.17, +2.20]
	AI InVivo	+0.44 ± 2.34	1.90 (5.75)	0.64 [0.38, 0.81]	0.64	−0.15 [−4.01, +3.71]
Body length (mm)	Manual	+0.87 ± 2.04	1.72 (5.02)	—	—	—
	Diagnocat	−0.01 ± 2.04	1.56 (4.70)	0.61 [0.26, 0.81]	0.66	+0.95 [−2.43, +4.32]
	AI InVivo	+0.52 ± 3.00	2.26 (7.13)	0.18 [−0.19, 0.50]	0.19	+0.35 [−6.10, +6.81]

Signed Δ = right − left. Δ agreement = ICC(2,1) of the automatic Δ with the manual Δ. Diagnocat *n* = 27–28; AI InVivo *n* = 31.

**Table 4:** Agreement of clinically relevant-asymmetry classification with manual tracing.

Parameter (Threshold)	System	Manual Prev.	Sens.	Spec.	κ	MAE	FN
Gonial angle (>4°)	Diagnocat	6/31 (19%)	0.00	1.00	0.00	2.05°	5
	AI InVivo	6/31 (19%)	0.17	0.96	0.17	1.76°	5
Ramus length (>3%)	Diagnocat	17/31 (55%)	0.67	0.67	0.33	2.31%	5
	AI InVivo	17/31 (55%)	**0.71**	**0.79**	**0.49**	1.50%	5
Body length (>3%)	Diagnocat	9/31 (29%)	0.44	0.83	0.29	1.57%	5
	AI InVivo	9/31 (29%)	0.44	0.68	0.12	1.92%	5

Asymmetry classified as |Δ| > 4° (angular) or AI% > 3% (linear). Sens., sensitivity; Spec., specificity; κ, Cohen’s kappa; MAE, mean absolute error of the asymmetry metric; FN, false negatives (truly asymmetric patients classified as symmetric). Thresholds are illustrative. All values are exploratory and descriptive only; the sample prevalence (e.g., 6/31, 19%, for the gonial angle) is insufficient for stable estimation of κ, sensitivity, or specificity, and the values are reported to characterise the direction of classification errors rather than to support diagnostic inference. The mandibular-plane parameter (1/31 positive case) is omitted for the same reason.

**Table 5:** Secondary analysis: agreement of bilateral incisor asymmetry (manual vs. Diagnocat; *n* = 28).

Parameter	|Δ| Manual	|Δ| Diagnocat	Δ-Agreement ICC [95% CI]	Δ Bias
Upper incisor U1–SN (°)	2.88°	2.56°	0.63 [0.35, 0.81]	+0.58
Lower incisor L1–MP (°)	3.80°	4.38°	0.91 [0.82, 0.96]	−0.11
Interincisal angle U1–L1 (°)	3.60°	4.00°	0.78 [0.58, 0.89]	−0.47

AI InVivo could not be evaluated for these parameters because it did not return left-side incisor values.

## Data Availability

The data presented in this study are available on request from the corresponding author. The data are not publicly available due to privacy and ethical restrictions relating to the CBCT imaging data of individual patients.
